# Supplementary material for: Clinicopathologic features and outcomes of bilateral lacrimal gland lesions
Source: PLoS One. 2024 Jul 3;19(7):e0305717. doi: 10.1371/journal.pone.0305717 (PMC11221706; doi:10.1371/journal.pone.0305717)
Supplement: Attachment — Submitted filename: Response to Reviewers.docx [file pone.0305717.s002.docx]

Dear editorial Professor: This is a rebuttal letter that responds to each point raised by the academic editor and reviewers. The black font is the revision suggestions by academic editors and reviewers, and the blue font is my answer. Thank you very much for your valuable suggestions on revision. Thank you.

1.Journal Requirements:

Answer:

My manuscript meets PLOS ONE's style requirements, including those for file naming.
Request for Permission to Publish Content under CC-BY License as an "Other" file with my submission.

Because Fig.1 has no effect on the result and outcome of this paper, and according to the modification suggestions, I have deleted Fig.1.

My tables had been inlcuded as part of my main manuscript in this submission and remove the individual files.

2. Editor Comments:
In my opinion and based on the reviewers` comments the manuscript needs revision. There are some points within the manuscript which must be corrected. The lack of clear Inclusion/Exclusion criteria with inclusion of previously treated patients are major drawbacks on the validity and reliability of the results and outcomes. The paper needs extensive English editing. Please find the attached files with reviewers` comments and carefully addressed all the suggestions.
Answer: In the materials and methods, I describe the inclusion criteria and exclusion criteria. According to the discussion of several ophthalmologis,I finally excluded patients of diagnostic lacrimal gland biopsy and patients with prior lacrimal gland medical and surgery intervention., and the results and outcomes are more validity and reliability. Furthermore,I asked a professional editing professor to help me with my English editing.

3.Reviewers' comments:

Reviewer #1: This study duplicates to a certain extent two other studies looking at bilateral lacrimal gland involvement by various diseases (both cited by these authors), but adds to that knowledge base by presenting a series from China.
There are quite a number of things that could be improved to make the paper more presentable and understandable. In general, the English is not good, and the paper needs extensive editing. Services are readily available online, and it is beyond the remit of the reviewers and editors of the journal to make those changes.

Answer: I made a lot of modifications to the paper as required and asked a professional editing professor to help me with my English editing.

Abstract - Results - "followed by dacryoadenitis" should have "non-specific" added before "dacryoadenitis"
- "inflammatory pseudotumor". This is not a diagnosis now supported by most specialists working in this field. Idiopathic orbital inflammation is a better term. How is this different to non-specific dacryoadenitis? What criteria were used to make this diagnosis and how was it separated from non-specific dacryoadenitis?

Answer: According to the diagnostic criteria of idiopathic orbital inflammation, inflammatory pseudotumor and dacryoadenitis in this paper are classified as idiopathic orbital inflammation. Therefore, inflammatory pseudotumor and dacryoadenitis in the paper were modified to idiopathic orbital inflammation (References:[Mombaerts](https://pubmed.ncbi.nlm.nih.gov/?term=Mombaerts%20I%5BAuthor%5D) I,[Bilyk](https://pubmed.ncbi.nlm.nih.gov/?term=Bilyk%20JR%5BAuthor%5D) JR, [Rose](https://pubmed.ncbi.nlm.nih.gov/?term=Rose%20GE%5BAuthor%5D) GE, [McNab](https://pubmed.ncbi.nlm.nih.gov/?term=McNab%20AA%5BAuthor%5D) AA, [Fay](https://pubmed.ncbi.nlm.nih.gov/?term=Fay%20A%5BAuthor%5D) A,[Dolman](https://pubmed.ncbi.nlm.nih.gov/?term=Dolman%20PJ%5BAuthor%5D) PJ,er al.Consensus on Diagnostic Criteria of Idiopathic Orbital Inflammation Using a Modified Delphi Approach. [JAMA Ophthalmol.](https://www.ncbi.nlm.nih.gov/pmc/articles/PMC5710199/) 2017 Jul; 135(7): 769-776. doi: [10.1001/jamaophthalmol.2017.1581](https://doi.org/10.1001%2Fjamaophthalmol.2017.1581)).
Results section of the paper:
- Demographics - what does "hormone therapy" mean?. Please specify what this is.
- Clinical presentation - what is "hypostasis"?
- Laboratory investigations - "the patient with MALT ....", and "The patient with elevated ...." Do you mean just one patient each time, or more than one?
- Radiological investigation - what does "long or equal on both T1 and T2" mean? Please clarify.
- Histopathology classification - what is the difference between dacryoadenitis (presumably non-specific) and inflammatory pseudotumor?
- Treatment - most common was surgery combined with corticosteroids. What exactly was entailed with the surgical part of this? Was it just incisional biopsy, or intentional debulking surgery? Incisional biopsy alone cannot be regarded as "treatment", but is rather an investigation.

Answer: Demographics- Hormone therapy involves patients who have used hormone for systemic diseases or eye diseases. According to the discussion of several ophthalmologis,I finally excluded patients of diagnostic lacrimal gland biopsy and patients with prior lacrimal gland medical and surgery intervention.
Clinical presentation – hypostasis was modified to lagophthalmos. Laboratory investigations – I mean just one patient each time. Radiological investigation - After consulting the professor of imaging, slightly long or equal on both T1 and T2 should be described as moderate signal intensities on MRI.
Histopathology classification - According to the diagnostic criteria of idiopathic orbital inflammation, inflammatory pseudotumor and dacryoadenitis in this paper are classified as idiopathic orbital inflammation. Therefore, inflammatory pseudotumor and dacryoadenitis in the paper were modified to idiopathic orbital inflammation (References:[Mombaerts](https://pubmed.ncbi.nlm.nih.gov/?term=Mombaerts%20I%5BAuthor%5D) I,[Bilyk](https://pubmed.ncbi.nlm.nih.gov/?term=Bilyk%20JR%5BAuthor%5D) JR, [Rose](https://pubmed.ncbi.nlm.nih.gov/?term=Rose%20GE%5BAuthor%5D) GE, [McNab](https://pubmed.ncbi.nlm.nih.gov/?term=McNab%20AA%5BAuthor%5D) AA, [Fay](https://pubmed.ncbi.nlm.nih.gov/?term=Fay%20A%5BAuthor%5D) A,[Dolman](https://pubmed.ncbi.nlm.nih.gov/?term=Dolman%20PJ%5BAuthor%5D) PJ,er al.Consensus on Diagnostic Criteria of Idiopathic Orbital Inflammation Using a Modified Delphi Approach. [JAMA Ophthalmol.](https://www.ncbi.nlm.nih.gov/pmc/articles/PMC5710199/) 2017 Jul; 135(7): 769-776. doi: [10.1001/jamaophthalmol.2017.1581](https://doi.org/10.1001%2Fjamaophthalmol.2017.1581)). Treatment - Surgery in this article is excisional lacrimal gland biopsy, which regarded as treatment.

Discussion -
- page 13 - the list of "mainly case reports" is misleading. There is a much larger literature than "mainly case reports" for diseases such as sarcoidosis, Erdheim-Chester disease, Sjögren's syndrome.
- page 14 - the paragraph starting "in addition, biopsy should be strongly ..." Why do you then suggest biopsy is NOT recommended in the "effectively treated"? Are some patients treated speculatively without a biopsy?
Answer:

page 13 -In the list of "mainly case reports"，I deleted sarcoidosis, Erdheim-Chester disease, Sjögren's syndrome and so on.

page 14 - According to the actual situation and clinical characteristics of this study, after discussion among ophthalmologists, pathologists and English teachers, it was found that there are some contradictions in the description here. Finally,“Therefore,we suggest that biopsy is not recommended for patients with bilateral lacrimal gland lesions who are effectively treated , but patients with poor long-term treatment or progressive worsening or recurrence are advised to undergo biopsy for timely diagnosis and treatment“ should be deleted.
Images - the histopathological images are of too low power and size to interpret, and add very little to the paper. I would delete them.

Answer:

Images -I deleted Fig.2.
Tables - Table 2, 3 and 4 (two last columns) suggest that percentages be added in brackets after each raw number. The raw numbers are relatively meaningless without the percentages.

Answer:

Tables – I added percentages in brackets after each raw number in Table 2, 3 ,4 and 5.

Reviewer #2: The authors stated in the Results section:
"In addition, 36 patients(27.9%) previously performed relevant treatment,including hormone therapy(n=16),excision of orbital mass (n=8) and submandibular gland(n=4) and eyelid mass (n=2)and conjunctival mass (n=1), fixation of lacrimal gland prolapse(n=2), double eyelid operation (n=1), radiotherapy (n=1) and chemotherapy(n=1)(Table 1)."
This is confusing since 16 patients had hormone therapy presumably for TED, however, the final results of the study didn't show TED cases. Also the authors didn't have well defined Inclusion/Exclusion criteria apart from "bilaterality".
The Bilaterality in some of the cases was identified only by Radiology, which was not clarified in the methodology.
The authors included patients with H/O hormone therapy, radiotherapy, and chemotherapy as well as previous excision of orbital mass.
All the above has negative impact on the validity and reliability of the results and outcome.
It might be wise to exclude those patients with previous therapy and to have the analysis repeated for the cases with primary bilateral LG lesions with no prior medical/surgical interference related to the original pathologies.
The manuscript can be considered after that.

Answer: In the materials and methods, I describe the inclusion criteria and exclusion criteria. According to the discussion of several ophthalmologis,I finally excluded patients of diagnostic lacrimal gland biopsy and patients with prior lacrimal gland medical and surgery intervention., and the results and outcomes are more validity and reliability. Furthermore, I analyzed and counted all the data again.

Hormone therapy involves patients who have used hormone for systemic diseases or eye diseases.

Patients with TED can be diagnosed according to the typical clinical characteristics and past medical history, but biopsy is basically not considered. Therefore, patients with TED do not meet the inclusion criteria.

A few patients presented with unilateral symptoms and signs at the time of seeing a doctor, but imaging examination revealed bilateral lacrimal gland lesions. Finally, it was confirmed by pathological examination. Therefore, in the materials and methods, We diagnosed bilateral lacrimal gland lesions utilizing a combination of clinical presentation, laboratory investigations, imaging findings and biopsy.

Reviewer #3: 1. Check reference no. 1. The format is not consistent with journal format.
2. Tables 1-5 were not included in the manuscript. Only the figures are available.
I can't relate to the discussion and conclusion. Answer: I have revised all the references according to **formatting references**. My tables are submitted as the individual files but my main manuscript did not included Tables 1-5. My tables had been inlcuded as part of my main manuscript in this submission. In the materials and methods, I describe the inclusion criteria and exclusion criteria. According to the discussion of several ophthalmologis,I finally excluded patients of diagnostic lacrimal gland biopsy and patients with prior lacrimal gland medical and surgery intervention., and the results and outcomes are more validity and reliability.
