# Supplementary material for: Clinicopathologic features and outcomes of bilateral lacrimal gland lesions
Source: PLoS One. 2024 Jul 3;19(7):e0305717. doi: 10.1371/journal.pone.0305717 (PMC11221706; doi:10.1371/journal.pone.0305717)
Supplement: Attachment — Submitted filename: Response to Reviewers.docx.docx [file pone.0305717.s003.docx]

Dear editorial Professor: This is a rebuttal letter that responds to each point raised by the academic editor and reviewers. The black font is the revision suggestions by academic editors and reviewers, and the blue font is my answer. Thank you very much for your valuable suggestions on revision. Thank you.

Reviewers' comments
Reviewer #4: This is just the review of a hospital with 129 patients only.
There is no new information in this article

Answer:

I have adequately addressed my modification in a previous round of review. In this study, We collected 113 cases of bilateral lacrimal gland lesions from January 2010 to December 2021. Their demographics, clinical features, the results of laboratory examinations, imaging presentations, histopathological diagnoses, treatments, and outcomes were analyzed in detail.

Reviewer #5: Although, according to the authors, no previous study has been published on the clinicopathological characteristics of bilateral lacrimal gland lesions biopsied in Asian populations, this paper has certain shortcomings that are markedly important. The quality of written English language text is at times suboptimal. The authors should specify some details about the referral West China Hospital of Sichuan University and the characteristics of the population it serves.
Furthermore, to enrich the content of the discussion, the authors could have compared the pattern of "bilateral" LG involvement with "unilateral" LG involvement data of their region.
The discussion implies that there are two main articles about the characteristics of bilateral LG involvement (by Huang et al in Australia and Tang et al in the US). A more detailed comparison of the findings of the present study with the two other similar case series would have been more informative.
Finally, as the authors have mentioned, the differences in the design of these studies prevent drawing conclusions from such comparison as, for instance, the IgG4 level testing may have been inconsistent among these studies. In this manuscript, only 31 of the 113 cases were tested for serum igG4 levels.

Answer:

I asked a professional editing professor to help me with my English editing again. The referral to West China Hospital of Sichuan University includes two forms: first, the patient is referred to West China Hospital of Sichuan University for histopathological examination after undergoing lacrimal gland resection surgery in another hospital, which does not meet the inclusion criteria and is excluded; Secondly, the patient was transferred to West China Hospital of Sichuan University for lacrimal gland resection surgery and histopathological examination through peer or online consultation, which met the inclusion criteria and was included in this study. This situation does not affect the conclusion of this paper.

In this article's discussion, I have added a comparison of data between bilateral lacrimal gland lesions and local lacrimal gland lesions, as well as a detailed comparison with the research results of Huang et al. in Australia and Tang et al. in the United States, such as clinical features, pathological diagnosis, treatment, etc.

In this study, the etiology of bilateral lacrimal gland lesions was determined by combining of clinical symptoms, results of laboratory investigations, imaging findings, and biopsy analysis rather than previous clinical diagnoses,and the level of IgG4 testing is consistent. In this study, although only 31 out of 113 cases underwent serum igG4 level testing, many patients underwent IgG4 immunostaining，The diagnosis of IgG4 ROD is correct. Therefore, the design of this study can draw conclusions in this paper.
