# Supplementary material for: Clinicopathologic features and outcomes of bilateral lacrimal gland lesions
Source: PLoS One. 2024 Jul 3;19(7):e0305717. doi: 10.1371/journal.pone.0305717 (PMC11221706; doi:10.1371/journal.pone.0305717)
Supplement: Attachment — Submitted filename: Response to Reviewers.docx.docx [file pone.0305717.s004.docx]

Dear editorial Professor: This is a rebuttal letter that responds to each point raised by the academic editor and reviewers. The black font is the revision suggestions by academic editors and reviewers, and the blue font is my answer. Thank you very much for your valuable suggestions on revision. Thank you.

Journal Requirements:
Please review your reference list to ensure that it is complete and correct. If you have cited papers that have been retracted, please include the rationale for doing so in the manuscript text, or remove these references and replace them with relevant current references. Any changes to the reference list should be mentioned in the rebuttal letter that accompanies your revised manuscript. If you need to cite a retracted article, indicate the article’s retracted status in the References list and also include a citation and full reference for the retraction notice.

Answer:

My reference list conforms to the reference format of plos one. All references were in correct format and did not cite a retracted article.

Reviewer #5: The revised paper is relatively improved and it has merit because of scarcity of similar studies conducted in its corresponding region (west China). However, the reader cannot find out how the "bilateral" lacrimal gland disorders compare to "unilateral" lacrimal gland involvement. So, I recommend the authors to present a very clear comparison between the two.

Answer:

In our area, I searched papers over the last 30 years and did not find a paper on unilateral lacrimal gland lesions. In other areas, I searched papers for the past 50 years and they were almost case reports. However, I just found a paper on unilateral lacrimal gland lesions. Therefore, in the discussion, I added a comparison between bilateral lacrimal gland lesions and unilateral lacrimal gland lesions. Correspondingly,in the references, I have added a reference([Gündüz](https://pubmed.ncbi.nlm.nih.gov/?term=G%C3%BCnd%C3%BCz+K&cauthor_id=14579116) K , [Shields](https://pubmed.ncbi.nlm.nih.gov/?term=Shields+CL&cauthor_id=14579116) CL, [Günalp](https://pubmed.ncbi.nlm.nih.gov/?term=G%C3%BCnalp+I&cauthor_id=14579116) I, [Shields](https://pubmed.ncbi.nlm.nih.gov/?term=Shields+JA&cauthor_id=14579116) JA. Magnetic resonance imaging of unilateral lacrimal gland lesions. Graefes Arch Clin Exp Ophthalmol. 2003 Nov;241 (11): 907-913. doi: 10.1007/ s00417-003-0748-z.)
